# Induction of Autophagy by Ursolic Acid Promotes the Elimination of *Trypanosoma cruzi* Amastigotes From Macrophages and Cardiac Cells

**DOI:** 10.3389/fcimb.2022.919096

**Published:** 2022-07-08

**Authors:** María Cristina Vanrell, Santiago José Martinez, Lucila Ibel Muñoz, Betiana Nebaí Salassa, Julián Gambarte Tudela, Patricia Silvia Romano

**Affiliations:** ^1^ Laboratorio de biología de Trypanosoma cruzi y la célula hospedadora, Instituto de Histología y Embriología de Mendoza, Instituto de Histología y Embriología de Mendoza-Consejo Nacional de Investigaciones Científicas y Técnicas (IHEM-CONICET)-Universidad Nacional de Cuyo, Mendoza, Argentina; ^2^ Facultad de Ciencias Médicas, Universidad Nacional de Cuyo, Mendoza, Argentina; ^3^ Facultad de Farmacia y Bioquímica, Universidad Juan Agustín Maza, Mendoza, Argentina; ^4^ Facultad de Odontología, Universidad Nacional de Cuyo, Mendoza, Argentina

**Keywords:** Chagas disease, *Trypanosoma cruzi*, amastigotes, autophagy, xenophagy, ursolic acid

## Abstract

Chagas disease, caused by the parasite *Trypanosoma cruzi*, is an infectious illness endemic to Latin America and still lacks an effective treatment for the chronic stage. In a previous study in our laboratory, we established the protective role of host autophagy *in vivo* during *T. cruzi* infection in mice and proposed this process as one of the mechanisms involved in the innate immune response against this parasite. In the search for an autophagy inducer that increases the anti-*T. cruzi* response in the host, we found ursolic acid (UA), a natural pentacyclic triterpene with many biological actions including autophagy induction. The aim of this work was to study the effect of UA on *T. cruzi* infection *in vitro* in the late infection stage, when the nests of intracellular parasites are forming, in both macrophages and cardiac cells. To test this effect, the cells were infected with *T. cruzi* for 24 h and then treated with UA (5–10 µM). The data showed that UA significantly decreased the number of amastigotes found in infected cells in comparison with non-treated cells. UA also induced the autophagy response in both macrophages and cardiac cells under the studied conditions, and the inhibition of this pathway during UA treatment restored the level of infection. Interestingly, LC3 protein, the main marker of autophagy, was recruited around amastigotes and the acidic probe LysoTracker localized with them, two key features of xenophagy. A direct cytotoxic effect of UA was also found on trypomastigotes of *T. cruzi*, whereas epimastigotes and amastigotes displayed more resistance to this drug at the studied concentrations. Taken together, these data showed that this natural compound reduces *T. cruzi* infection in the later stages by promoting parasite damage through the induction of autophagy. This action, in addition to the effect of this compound on trypomastigotes, points to UA as an interesting lead for Chagas disease treatment in the future.

## Introduction

Chagas disease (CD) or American trypanosomiasis is caused by the protozoan parasite *Trypanosoma cruzi*. The WHO estimates that 8 to 10 million people are infected worldwide, mostly in Latin America, where the disease is endemic. CD is also one of the so-called neglected tropical diseases because of the low attention that governments and industries gave to these illnesses in the past. Benznidazole (BNZ) and nifurtimox are currently the only two drugs approved for the treatment of CD. Although several laboratories are working in the search for new therapies, the lack of effective drugs for the chronic stage is still unsolved.

*T. cruzi* has a biphasic biological cycle that develops in both the insect vector and the mammalian hosts. In the latter, *T. cruzi* behaves as an obligate intracellular parasite, interacting with compartments from the vesicular transport pathways to invade the host cell and establish its replicative niche (Nunes et al., 2013; Dias, 2017; Guarner, 2019). *T. cruzi* is capable of infecting several classes of host cells. Macrophages are the first line of defense during *T. cruzi* invasion. Macrophages may either suppress *T. cruzi* replication or provide a favorable environment where the parasite can reproduce and be distributed to other tissues within the body (Peluffo et al., 2004; Holzmuller et al., 2018). However, cardiac cells are one of the main targets of *T. cruzi*, in which the parasite establishes its replicative niche to form the amastigote nests that are present in the heart of the chronic patients (De Alba-Alvarado et al., 2020).

Ursolic acid (UA) is a naturally occurring pentacyclic triterpene with many biological properties. This compound is widely distributed in nature, in aromatic plants such as rosemary, basil, oregano, and eucalyptus, as well as medlar fruits, apple skin, and coffee seeds. It has been shown that UA displays anti-inflammatory, antioxidant, and anticancer activities (Chen et al., 2020; Khwaza et al., 2020). Moreover, UA exerts potent antiviral action (Tohmé et al., 2019) and has an antiprotozoal effect against species of *Leishmania* and *Plasmodium* (Bilbao-Ramos et al., 2020; Son and Lee, 2020). In *T. cruzi*, UA displayed trypanocidal activity on epimastigotes at a concentration of 100 μM (Uchiyama et al., 2006). Oral administration of UA reduces the parasitemia peaks in mice infected with *T. cruzi* Y strain in an acute model of infection (da Silva Ferreira et al., 2013). Interestingly, UA has been described as an autophagy inducer in cancer cells (Deng et al., 2019; Lin et al., 2020). UA inhibited proliferation and induced autophagy and apoptosis in several cancer models, including breast carcinoma, melanoma, leukemia, hepatoma, and prostate cancer (Pathak et al., 2007; Shanmugam et al., 2011; Leng et al., 2013; Zhao et al., 2013). In a primary brain tumor model, UA activated autophagy by modulation of the calmodulin-dependent kinase protein kinase (CaMMK)/AMPK/mTOR through the induction of ER stresses and Ca^2+^ release (Shen et al., 2014). This mechanism was also observed in gemcitabine-resistant human pancreatic cancer cells treated with UA (Deng et al., 2019; Lin et al., 2020). Other works have also demonstrated the effects of different natural or synthetic compounds on the intracellular stages of protozoan parasites (Mallick et al., 2011; Yousuf et al., 2015; Sultana et al., 2018; Mukherjee et al., 2020). These studies evaluated the effect of compounds against *Leishmania* spp. *in vitro* and *in vivo*, showing actions on both parasitic targets (mitochondrion, antioxidant enzymes, etc.) and increasing the innate immune response of the infected host.

In a previous study, we demonstrated that the deficiency of autophagy in the Beclin-1 heterozygous knockout mice exacerbates *T. cruzi* infection. This effect correlated with a higher parasite load observed in both peritoneal macrophages obtained from these mice and in macrophages obtained from wild-type (WT) animals treated with autophagy inhibitors (Casassa et al., 2019). In agreement with these data, there is evidence that *T. cruzi* is able to induce an increase in the expression of LC3-II, as well as the formation of autophagosomes and autolysosomes in macrophages, and that the pharmacological inhibition of the autophagy machinery impairs the ability of macrophages to control amastigote replication (Matteucci et al., 2019). Given that autophagy plays a protective role in *T. cruzi* infection *in vivo* and that UA induces autophagy in different cellular models, we proposed that UA treatment impairs *T. cruzi* infection due to its action on autophagy on infected cells. To test this hypothesis, we studied the effect of UA in the intracellular cycle of *T. cruzi* in both macrophages and cardiac cells, two main cells targeted by *T. cruzi* during the course of human infection. Moreover, considering that the chronic stage of infection is still difficult to cure because of the presence of intracellular parasites hidden in the tissues, these studies were conducted in the late stages of infection, when amastigotes are replicating in the cytosol of host cells.

## Methods

### Reagents

Dulbecco’s Modified Eagle Medium (DMEM), penicillin, and streptomycin were obtained from Gibco BRL/Life Technologies (Carlsbad, CA, USA). The polyclonal rabbit anti-LC3 antibody and UA were purchased from Sigma-Aldrich (St. Louis, MO, USA). The polyclonal mouse anti-β TUBULIN was obtained from Developmental Studies Hybridoma Bank. Serum from *T. cruzi*-infected C57 mouse was obtained and used for *T. cruzi* detection. The secondary antibody Cy3-conjugated anti-Goat IgG was purchased from Jackson ImmunoResearch Laboratories (West Grove, PA, USA). The secondary antibody Alexa 488 was obtained from Thermo Fisher (Waltham, MA, USA). The DNA marker Hoechst 33342 and LysoTracker Red were purchased from Life Technologies. The fetal bovine serum (FBS) was purchased from Natocor (Cordoba, Argentina). Red DQ-BSA was obtained from Invitrogen (Carlsbad, CA, USA). The nitrocellulose and chemiluminescence detection kit was from Amersham (Pittsburgh, PA, USA), and the *In Situ* Cell Death Detection Kit was purchased from Roche (Basel, Switzerland).

### Cell Culture

RAW 264.7 (murine macrophages) and H9C2 (rat myoblast) cells were maintained in flasks in DMEM supplemented with 10% FBS and antibiotics in a 5% CO_2_ atmosphere.

### Preparation of Bone Marrow Macrophages

Bone marrow was obtained from the femur bones of C57BL/6J WT and KD (Beclin-1 heterozygous knockout mice, Beclin-1 ±) mice and resuspended in cold DMEM, containing 40 μg/ml of gentamicin, following standard procedures. These bone marrow progenitor cells were recovered in 100-mm plates, containing 10% FBS, 40 µg/ml gentamicin, 2 mM of l-glutamine, and a conditioned medium derived from 30% L929 cell culture, for 4 days. Then, they were washed, and the same medium was added for an additional 6 days. Finally, the cells were typed by flow cytometry (BD FACSAria III, BD Biosciences, San Jose, CA, USA) using the markers F480 (PerCP) and CD11b (APC) to confirm the identity of macrophages (**Supplementary Figure 1**).

### Quantification of *Trypanosoma cruzi* Infection

RAW macrophages, bone marrow macrophages (BMMs) from C57 WT and KD mice (Beclin-1 heterozygous knockout mice, deficient in the autophagic pathway), and H9C2 cells were infected with 10 trypomastigotes (multiplicity of infection (MOI) = 10) of Y strain (for Indirect Immunofluorescence analysis, IIF) or Y-GFP (for Western blotting study) per cell for 24 h. After being washed to eliminate the parasites that did not infect the cells, a fresh medium was added with or without 5 or 10 µM of UA and incubated for 24, 48, or 72 h. Cells were then processed to detect *T. cruzi* amastigotes by Western blotting or indirect immunofluorescence.

For indirect immunofluorescence, the cells were fixed with 4% paraformaldehyde in phosphate-buffered saline (PBS) for 20 min at room temperature, then washed with PBS, blocked with 50 mM of NH_4_Cl, and permeabilized with 1% saponin in PBS containing 1% bovine serum albumin (BSA). Samples were then incubated with an anti-*T. cruzi*-specific antibody followed by incubation with an anti-rabbit secondary antibody conjugated with Cy3 fluorophore or Alexa 488 and then mounted with Mowiol 4-88 reagent containing Hoechst 33342. Samples were then examined by confocal microscopy using an Olympus Confocal FV1000 microscope (Tokyo, Japan) and processed with the program FV10-ASW 1.7 for further analysis.

For Western blotting studies, infected cells were lysed with sample buffer, and protein samples were run on a 10% polyacrylamide gel and transferred to nitrocellulose membranes. The membranes were then blocked overnight (ON) in Blotto at 4°C (5% non-fat milk, 0.1% Tween 20, and PBS), washed twice with PBS, and incubated with a primary antibody anti-*T. cruzi* (1:800 dilution) followed by a peroxidase-conjugated secondary antibody (1:5,000 dilution). The primary anti-TUBULIN antibody (1:300 dilutions) was used to detect TUBULIN as a loading control. The bands were detected by using an enhanced chemiluminescence detection kit (Amersham, Piscataway, NJ, USA; RPN2109) followed by the detection of signals in a Fujifilm LAS-4000.

### Detection of LC3 Protein

Autophagy was induced by amino acid starvation. RAW macrophages and H9C2 cells grown in 6- or 24-well plates were washed three times with PBS and incubated with a control medium or Earle’s balanced salt solution (starvation medium) at 37°C for 2 h in the presence or absence of drugs (100 nM of wortmannin or 10 µM of UA).

For detection by immunofluorescence, the cells were fixed with 4% paraformaldehyde in PBS for 15 min at room temperature, washed with PBS, and blocked with 50 mM of NH_4_Cl. Subsequently, cells were permeabilized with 1% saponin in PBS containing 1% BSA, followed by incubation with an anti-LC3-specific antibody. After incubation with a Cy3-conjugated anti-rabbit antibody, each sample was mounted with Mowiol 4-88 reagent and examined by confocal microscopy using an Olympus Confocal FV1000 microscope (Japan). Images were then processed with the program FV10-ASW 1.7. The percentage of cells was determined with more than 5 or 10 LC3 dots/cell. Confocal images of 10 random fields were quantified, representing around 100 cells per experiment. Data are presented as mean values, and error bars indicate the SEM from at least three independent experiments. Statistical calculations were made using Kyplot statistical software.

Western blotting analysis was performed as explained above. Samples were run on a 12.5% polyacrylamide gel, transferred to nitrocellulose membranes, and incubated with a primary antibody anti-LC3 (1:800 dilution) followed by a peroxidase-conjugated secondary antibody (1:10,000 dilution). Anti-TUBULIN (1:300 dilution) was used to detect TUBULIN as a loading control. The corresponding bands were detected using an enhanced chemiluminescence detection kit (Amersham, RPN2109) in a Fujifilm LAS-4000.

### DQ-BSA Labeling

RAW macrophages overexpressing GFP-LC3 grown in 24-well plates were washed three times with PBS and incubated with a control medium at 37°C for 2 h in the presence or absence of 10 µM of UA. Thirty minutes before the end of the reaction, 10 µg/ml of DQ-BSA was added. This compound was used to identify degradative compartments by fluorescence microscopy. Cells were then fixed with 4% paraformaldehyde in PBS for 15 min at room temperature, washed with PBS, blocked with 50 mM of NH_4_Cl in PBS, and mounted with Mowiol 4-88 reagent containing Hoechst 33342 to label DNA. Samples were examined by confocal microscopy, using an Olympus Confocal FV1000 microscope (Japan), and processed with the program FV10-ASW 1.7.

### LysoTracker Labeling

RAW macrophages overexpressing GFP-LC3 grown in 24-well plates were washed three times with PBS and incubated with a control medium at 37°C for 2 h in the presence of LysoTracker Red and the presence or absence of 10 µM of UA. The cells were then fixed with 3% paraformaldehyde in PBS for 15 min at room temperature, washed with PBS, and blocked with 50 mM of NH_4_Cl in PBS. They were mounted with Mowiol 4-88 reagent containing Hoechst 33342, examined by confocal microscopy, using an Olympus Confocal FV1000 microscope (Japan), and processed with the program FV10-ASW 1.7.

In addition, RAW macrophages were infected with trypomastigotes of the Y strain with 10 parasites per cell (MOI 10), for 24 h. Then they were washed to eliminate the parasites that did not cause infection, and a fresh medium was added with or without 10 µM of UA for 24 additional hours. Two hours before the end of the reaction, LysoTracker Red was added. Then the cells were fixed with 3% paraformaldehyde in PBS for 15 min at room temperature, washed with PBS, and blocked with 50 mM of NH_4_Cl in PBS. They were mounted with Mowiol 4-88 reagent containing Hoechst 33342 and examined by confocal microscopy, using an Olympus Confocal FV1000 microscope (Japan), and processed with the program FV10-ASW 1.7.

### Epimastigote Viability

Epimastigotes of the Y strain were grown in Diamond medium alone or with the addition of 5, 10, 12.5, 25, 50, and 100 µM of UA at 28°C for 24 h. A small sample was extracted, and live epimastigotes were counted in a Neubauer chamber. To calculate the inhibitory concentration 50 (IC50) and the graphs, the Microsoft Excel program was used. Some of these parasites were analyzed by transmission electron microscopy (TEM), as explained above.

### Trypomastigote Viability

Trypomastigotes (1,000,000 for each condition) of the Y strain were incubated for 0, 1.5, 3.5, 6, and 24 h at 4°C in DMEM alone or more than 10 or 50 µM of UA. At different times, a small sample was extracted, and live trypomastigotes were counted in a Neubauer chamber. To calculate the half maximal effective concentration (EC50) and the graphs, the Microsoft Excel program was used.

### Amastigote Viability

RAW macrophages and H9C2 cells were infected with trypomastigotes of the Y strain with 10 parasites per cell (MOI 10) for 24 h. They were then washed to eliminate the parasites that did not cause infection, and a fresh medium was added without or with 10 µM of UA for 24 additional hours. Subsequently, the instructions of the manufacturer of the *In Situ* Cell Death Detection Kit were followed.

### Transmission Electron Microscopy

RAW macrophages and H9C2 cells were infected with trypomastigotes of the Y strain with 10 parasites per cell (MOI 10) for 24 h. They were then washed to eliminate the parasites that did not cause infection, and a fresh medium was added with or without 10 µM of UA for 24 additional hours. After that, cells were fixed with 2.5% glutaraldehyde (Pelco International, Fresno, CA, USA) in PBS at 10°C and processed by the Servicio de Preparación de Muestras de Microscopía Electrónica, STAN 3371IHEM-CONICET.

Briefly, each sample was washed in the same buffer, post-fixed in 1% OsO for 1 h at room temperature, dehydrated in a graded acetone solution series, and embedded in low viscosity epoxy resin (Pelco International). Then, ultrathin sections with interference color gray were cut by ultramicrotome Leica Ultracut R, mounted on grids, and stained with uranyl acetate and lead citrate (Reynolds 1963). Grids were examined under a Zeiss 900 electron microscope, with a Gatan digital camera (model Orius SC 1000).

## Results

### Effect of Ursolic Acid on the Late Stages of *Trypanosoma cruzi* Infection *In Vitro*

**Figure 1:**
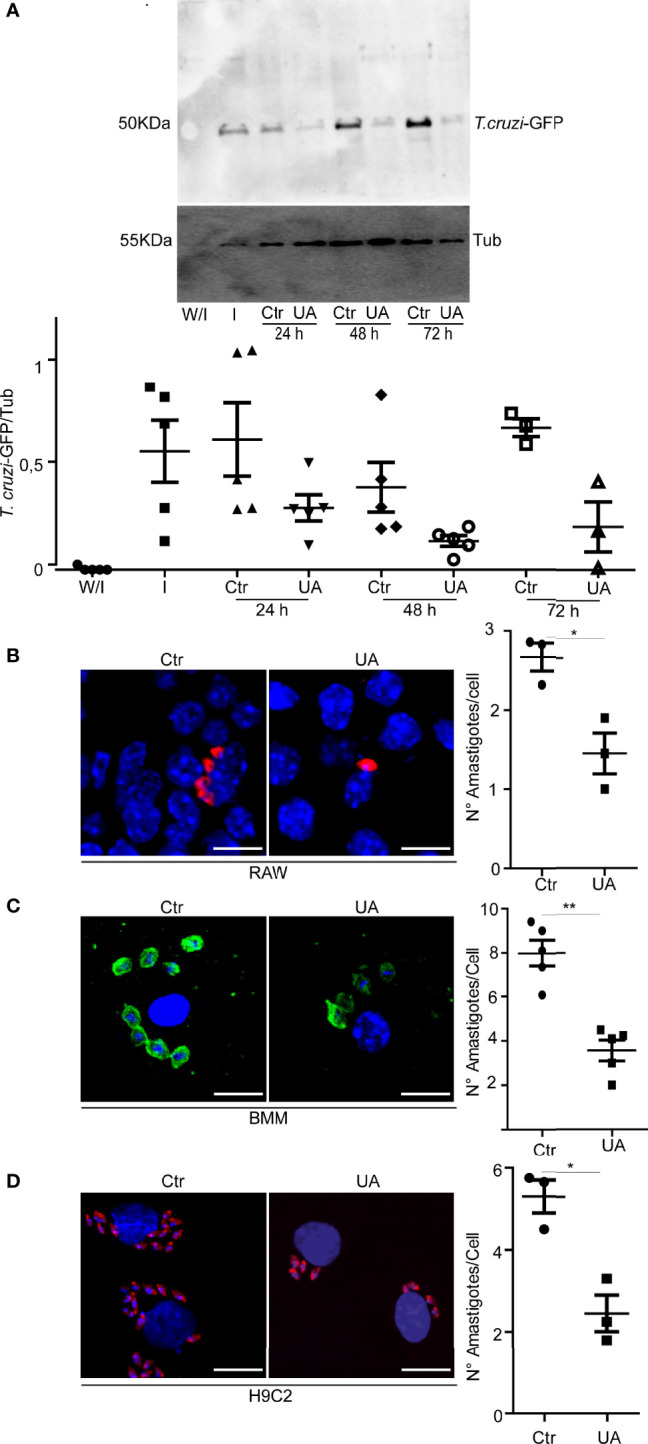
Effect of UA on the number of intracellular amastigotes. RAW macrophages, BMM, or H9C2 cells infected with *Trypanosoma cruzi* Y or Y-GFP strain (MOI = 10) for 24 h and then treated for additional 24, 48, or 72 h under different conditions. **(A)** Representative immunoblots are depicted. Densitometry was performed using NIH ImageJ. We calculated the GFP/TUBULIN. Data represent the mean ± SEM of five independent experiments. **(B)** Confocal images showing amastigotes of *T. cruzi* (red) in RAW macrophages under the indicated conditions. Scale bars, 10 μm. Quantification of the number of amastigotes per cell. Data represent the mean ± SEM of three independent experiments (number of counted cells ≈100). *p < 0.05, Tukey’s test. **(C)** Confocal images showing amastigotes of *T. cruzi* (Green) in BMM under the indicated conditions; here we used 5 µM of UA. Scale bars, 10 μm. Quantification of the number of amastigotes per cell. Data represent the mean ± SEM of at least three independent experiments (number of counted cells in each experiment ≈ 100). **p < 0.01, Tukey’s test. **(D)** Confocal images showing amastigotes of *T. cruzi* (red) H9C2 under the indicated conditions at 48 h of treatment. Scale bars, 10 μm. Quantification of the number of amastigotes per cell at 24 h of treatment. Data represent the mean ± SEM of at least three independent experiments (number of counted cells ≈ 100). UA, ursolic acid; BMM, bone marrow macrophage; MOI, multiplicity of infection.

To analyze the possible effect of UA on the infection of *T. cruzi*, we infected macrophages derived from BMM, RAW macrophages, and H9C2 cells (rat cardioblasts) with trypomastigotes of the *T. cruzi* Y or Y-GFP strain for 24 h and treated them with 5 or 10 µM of UA for an additional time of 24, 48, or 72 h. Both cell types are important in CD since macrophages are the first line of defense and muscle-derived cells, such as H9C2 cells, are the main target cells with which *T. cruzi* displays a great affinity. Subsequently, we quantified the number of amastigotes present in the treated cells by either Western blotting or confocal microscopy in comparison with infected cells maintained in the control medium (**Figure 1**). The number of amastigotes in RAW cells was studied at different times of treatment by detection of the GFP protein present in the parasites with an anti-GFP-specific antibody by Western blotting. A marked reduction in the infection was observed in the conditions with UA (10 μM) in comparison with the control conditions (**Figure 1A**). Because the effect of the UA on the number of amastigotes was observed from 24 h, further studies were carried out at this time.

Lower detection of *T. cruzi* amastigotes was also observed by confocal microscopy in RAW cells, as well as in BMM treated with 10 or 5 μM of UA for 24 h. Quantitative data showed that UA significantly reduced the number of amastigotes/cells in both cell types (**Figures 1B, C**). A similar effect on the content of amastigotes was observed in H9C2 cells (**Figure 1D**).

Due to the known anticancer effect of UA, prior to these studies, we demonstrated that UA is not toxic in RAW macrophages or H9C2 cells at the concentrations studied. With the use of the AlamarBlue reagent, similar cell vitality was observed in cells treated with 10 μM of UA in comparison with untreated controls for both macrophages and cardiac-derived cells (**Supplementary Figure 2**). Taken together, these data demonstrated that UA impairs the intracellular cycle of *T. cruzi*, resulting in a significant reduction in the number of amastigotes present in the host cell cytoplasm at late times of infection.

### Ursolic Acid Induces Autophagy and Xenophagy of *Trypanosoma cruzi* Amastigotes

In a previous study, we demonstrated that autophagy plays a protective effect in a mouse model of *T. cruzi* infection. We observed that mice deficient in autophagy (heterozygous knockout for *Beclin-1* gene) (Qu et al., 2003; Haspel et al., 2011) developed a more aggressive infection characterized by higher parasitemia values and earlier death than did autophagy-competent mice. This study also showed that macrophages from deficient mice or WT macrophages treated with autophagy inhibitors displayed a lower capacity to clear amastigotes by the process of xenophagy (Casassa et al., 2019). Xenophagy, the process of capture and degradation of intracellular pathogens, is a class of selective autophagy that belongs to the repertoire of the innate immune responses activated in phagocytic cells against intracellular microorganisms (Sharma et al., 2018). Since UA was previously shown to be an autophagy inducer (Leng et al., 2016), next, we asked whether the action of this compound in the reduction of amastigotes was produced by xenophagy.

**Figure 2:**
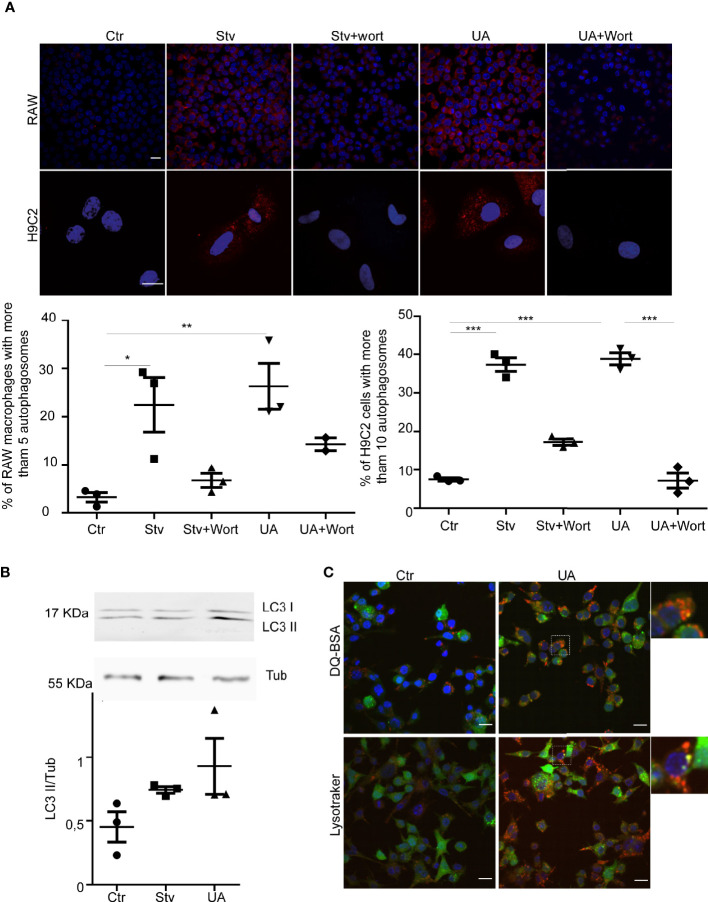
Ursolic acid (UA) stimulates autophagy in RAW macrophages and H9C2 Cells. RAW macrophages or H9C2 cells were incubated for 2 h in control or starvation medium (Stv) or in control medium supplemented with 10 µM of UA alone (UA) or in the presence of 100 nM of wortmannin (UA+Wort) or starvation medium (Stv) in the presence of 100 nM of wortmannin (Stv+Wort) as indicated in *Methods*. **(A)** Images show the LC3 (red) distribution in the indicated conditions. Scale bars, 10 μm. Graph represents the percentage of cells with more than 5 dots (RAW macrophages) or 10 dots per cell (H9C2 cells) in each condition. Data represent the mean ± SEM of three independent experiments (number of counted cells in each experiment ≈ 100). *p < 0.05, **p < 0.01. Tukey’s test. **(B)** Top panel: representative immunoblot of three experiments corresponding to LC3 detection is depicted. Bottom panel: quantification of the LC3II/Tub ratio. Data are representative of three independent experiments. **(C)** RAW macrophages overexpressing GFP-LC3 were grown in control medium in the presence or absence of 10 µM of UA (UA) for 2 h. Confocal images depict GFP-LC3 and DQ-BSA or LysoTracker distribution under the indicated conditions. Scale bars, 10 μm.

To test this hypothesis, we first studied the possible effect of UA on the autophagy response of RAW and H9C2 cells. Cells were treated with UA for 2 h, and the presence of autophagosomes was analyzed by detection of endogenous LC3 protein by IIF followed by confocal microscopy. Cells subjected to conditions of induction (starvation (Stv)) or inhibition (Stv+Wort) of autophagy were added as controls (see details in *Methods*). As shown in **Figure 2A**, a different number of autophagosomes formed in each condition and according to the class of cell assayed. We next quantified the percentage of cells with more than 5 (for RAW cells) or 10 (for H9C2 cells) autophagosomes/cell, indicative of an active autophagy response, as previously shown (Vanrell et al., 2013). As expected, the values significantly increased in cells under starvation in comparison with control cells (Ctr) and decreased in the presence of wortmannin, a classic autophagy inhibitor. Interestingly, as with starvation, the treatment with UA increased the number of autophagosomes, which also diminished in the presence of wortmannin. To confirm these data, we next detected the endogenous LC3 by Western blotting and observed an increment in the level of LC3-II in the presence of UA (**Figure 2B**). Together, these data showed that UA treatment produced a significant increment in the number of autophagosomes in both RAW macrophages and H9C2 cells. Both inductions of autophagy and inhibition of autophagy degradation resulted in an increase in the number of autophagosomes. Therefore, to confirm the action of UA on autophagy, we next treated RAW cells overexpressing GFP-LC3 with UA and then incubated them with DQ-BSA and LysoTracker, markers of hydrolytic and acidic compartments, respectively, to study the nature of autophagosomes. As shown in **Figure 2C**, under UA treatment, many autophagosomes decorated with GFP-LC3 were also stained with DQ-BSA or LysoTracker, indicating their autolysosomal nature and confirming that treatment with UA induced a functional autophagy response.

**Figure 3:**
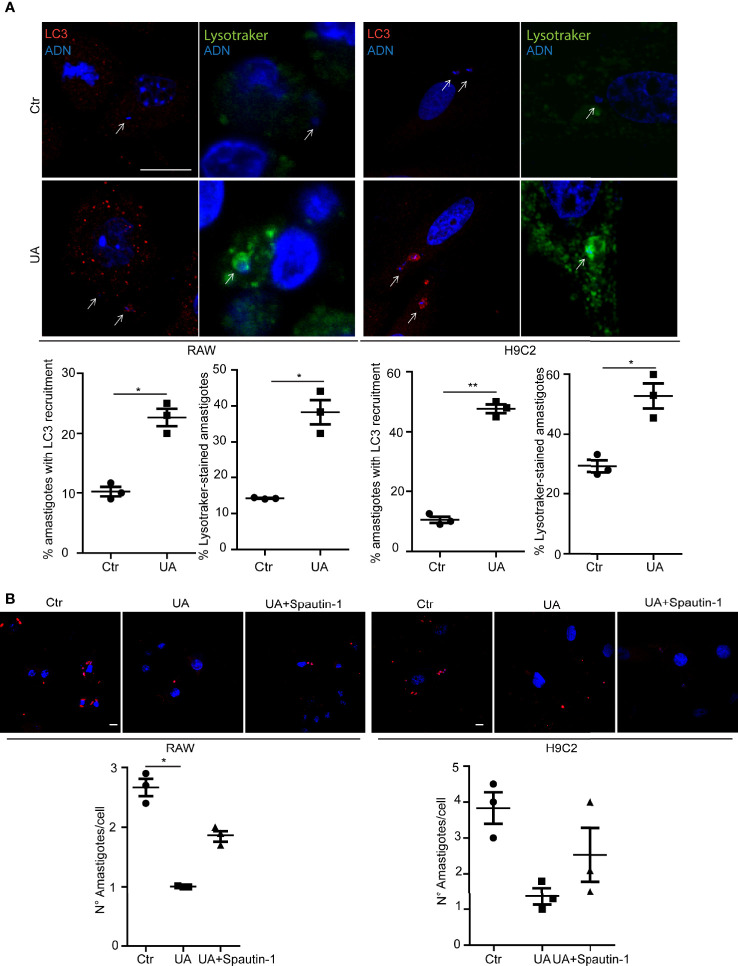
Study of the *Trypanosoma cruzi* xenophagy. RAW macrophages or H9C2 cells infected with *T. cruzi* strain Y (MOI = 10) for 24 h and then treated for additional 2 or 24 h in different conditions. **(A)** RAW macrophages or H9C2 cells infected with *T. cruzi* Y strain for 24 h and then treated for an additional 2 h under different conditions. Confocal images depicting recruitment of LC3 (red) detected by indirect immunofluorescence or LysoTracker (Green) to *T. cruzi* amastigotes. Scale bars, 10 μm. White arrows point to amastigotes. The graphs show the percentage of the mean ± SD of three independent experiments on the recruitment of LC3 or LysoTracker in the different conditions. **p < 0.01. Tukey’s test. **(B)** RAW macrophages or H9C2 cells infected with *T. cruzi* Y strain (MOI = 10) for 24 h and then treated for an additional 24 h in different conditions, control, 10 µM of UA, or 10 µM of UA with Spautin-1. Confocal images show *T. cruzi* amastigotes (red) under different conditions. The graphs represent the mean ± SEM of the number of amastigotes per cell under the different conditions. MOI, multiplicity of infection.

Next, we studied the possible participation of autophagy in the elimination of amastigotes. RAW macrophages and H9C2 cells were infected with trypomastigotes of *T. cruzi* Y strain for 24 h and then treated with a control medium alone or with 10 µM of UA for an additional 24 h. After fixation, cells were processed to detect the possible recruitment of LC3 protein to amastigotes and the presence of LysoTracker. Interestingly, both markers were found in amastigotes under control or UA-treated conditions. However, under UA conditions, the percentage of amastigotes with LC3 recruited was greater than in the control in both cell types. Similar differences were obtained when LysoTracker staining was quantified (**Figure 3A**). These data suggest that reduction in the number of amastigotes in UA-treated cells is mediated by an increment in amastigote xenophagy in these cells. To confirm that UA-induced autophagy was responsible for the clearance of amastigotes in the host cell cytoplasm, we performed similar experiments in the presence of the autophagy-specific PI3K inhibitor, Spautin-1 (Correa et al., 2014). RAW macrophages or H9C2 cells were infected with trypomastigotes of *T. cruzi* for 24 h and treated with 10 or 10 µM of UA in the presence of 10 µM of Spautin-1 for an additional 24 h. Cells were then fixed and processed to detect amastigotes by IIF by using an anti-*T. cruzi*-specific antibody. Notably, the inhibition of the autophagy pathway by Spautin-1 reversed the effect of UA on the number of amastigotes (**Figure 3B**). These data confirm that UA induces autophagy, and as a consequence, it promotes the elimination of amastigotes from the cytoplasm of host cells.

In another set of experiments, we performed primary cultures of BMM obtained from *Beclin-1* heterozygous knockout mice (KD) and studied the level of infection in the presence of UA in comparison with cells obtained from WT animals. In agreement with our previous results (Casassa et al., 2019), the number of amastigotes in cells that displayed reduced autophagy was higher than that in the BMM WT. Treatment of control cells with UA reduced the number of amastigotes by ≈50%, as expected. In contrast, the same treatment in KD cells produced a partial effect due to the low autophagy response displayed by these cells (**Supplementary Figure 3**).

### Exploring a Direct Action of Ursolic Acid Against *Trypanosoma cruzi*

To further study the possibility of a direct cytotoxic action of UA on *T. cruzi*, we analyzed the effect of this compound on the two replicative stages of this parasite, epimastigotes, and amastigotes, as well as in the infective trypomastigote stage.

**Figure 4:**
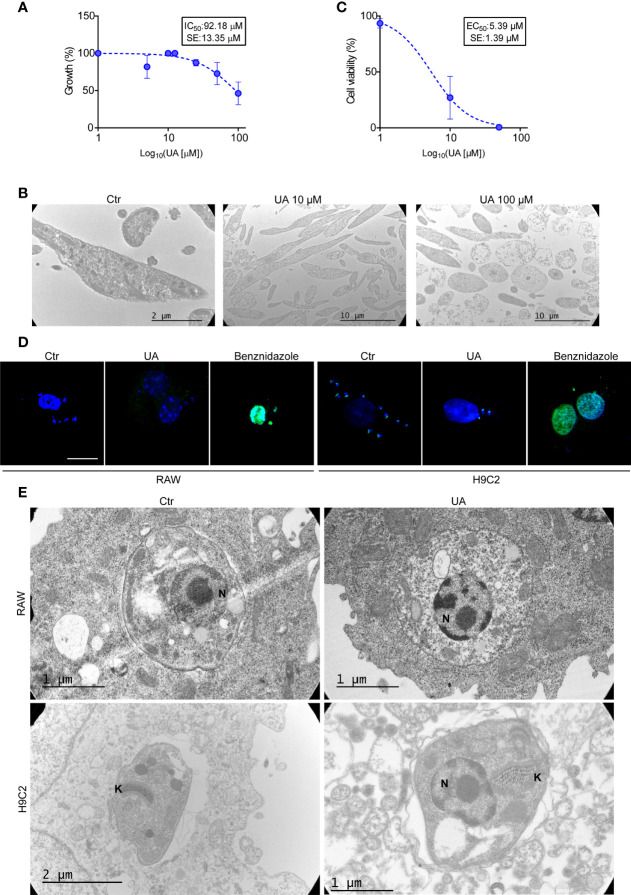
Direct action of UA on different stages of *Trypanosoma cruzi*. **(A)** Epimastigotes were grown at different concentrations of UA for 24 h at 28°C. The graph shows the quantification of IC50. **(B)** Transmission electron microscopy images of epimastigotes treated under different conditions for 24 h. **(C)** The trypomastigotes were treated at different concentrations of UA for 24 h at 4°C. The graph shows the quantification of the EC50. **(D)** RAW macrophages and H9C2 cells were infected with *T. cruzi* trypomastigotes Y strain (MOI = 10) for 24 h and then treated for an additional 24 h under the different conditions. Subsequently, we evaluated apoptosis of the amastigotes and/or host cells using the TUNEL assay. White arrows point to amastigotes. **(E)** RAW macrophages and H9C2 cells were infected with *T. cruzi* trypomastigotes Y strain (MOI = 10) for 24 h and then treated for an additional 24 h under the different conditions. Transmission electron microscopy images show amastigotes inside the host cell. UA, ursolic acid; MOI, multiplicity of infection.

First, we studied the cell viability of epimastigotes and trypomastigotes by treating the parasites with increased concentrations of UA for 24 h. As shown in **Figures 4A, C**, while the IC50 for epimastigotes was 101.02 ± 9.91 µM, the EC50 for trypomastigotes was 5.39 ± 0.02. The cytotoxic effect on epimastigotes was confirmed by the observation of damaged parasitic cells by TEM at 100 μM without changes in the cellular morphology at lower concentrations (**Figure 4B**). These data indicate that UA showed high toxicity for *T. cruzi* trypomastigotes but not for epimastigotes.

Next, we studied the direct action of UA on amastigotes, the replicative stage of *T. cruzi* in mammalian cells. RAW macrophages and H9C2 cells were infected with *T. cruzi* trypomastigotes Y strain for 24 h and then incubated in a control medium alone or with the addition of 10 µM of UA or 50 nM of BNZ, which was used as a positive control of death. Using the TUNEL reagent for fluorescence microscopy, we evaluated the apoptosis of amastigotes by the green fluorescence emitted by the nucleus of dead parasites. As shown in **Figure 4D**, apoptotic parasites were produced under BNZ treatment in both RAW and H9C2 cells, while controls and UA-treated cells were negative for TUNEL staining, indicating the absence of apoptosis in these conditions. Note that the kinetoplasts of live amastigotes can be stained with this reagent, but the nuclei cannot, as these are stained when the amastigote dies by apoptosis (De Souza et al., 2010). However, in cells treated with BNZ, positive apoptotic nuclei were observed in amastigotes and the host cell, evidencing the cytotoxic action of this drug even in the mammalian cells (**Figure 4D**).

To confirm the low cytotoxic effect of UA on amastigotes, we further studied the ultrastructure of amastigotes developed in the host cells by TEM. No structural differences were observed in the amastigotes present in cells under a control medium or treated with UA (**Figure 4E**). These data showed that UA has a direct cytotoxic action on trypomastigotes of *T. cruzi* but not on the replicative stages, epimastigotes, and amastigotes and confirm that the host cell autophagy is required for the elimination of amastigotes from the host cell.

## Discussion

One of the main challenges in the search for new drugs for the treatment of CD is to find more effective compounds for the chronic stage than the current therapies. The persistence of amastigote nests in the tissues of chronic patients induces an immune response that further produces the complications of this disease, evidencing the necessity of a trypanocidal action that clears the tissue parasitosis (Monteon-Padilla et al., 2001; Inst et al., 2011). Many strategies are being studied to reach this goal: inhibition of ergosterol synthesis, impairment of the action of virulence factors such as cruzipain, inhibition of the parasite redox metabolism, and other strategies that target vital processes for *T. cruzi*. Other less explored strategies include the regulation of the host immune responses to enhance their antiparasitic activity. This study was focused on the latter paradigm. Based on evidence showing the key role of autophagy in the control of *T. cruzi* infection *in vitro* and *in vivo* (Márquez et al., 2018; Casassa et al., 2019; Matteucci et al., 2019), we decided to search for a compound that increases the autophagy response of host cells and, consequently, interferes with the *T. cruzi* intracellular cycle. Since not all inducers of autophagy can be administered to patients due to their toxic action or because they display other unwanted effects, we selected UA. This natural compound has many biological actions including autophagy induction on different tumor-derived cells (Deng et al., 2019).

The first results of this work demonstrated that UA reduced the content of amastigotes in macrophages, as well as in cardiac cells, without affecting host cell viability, as shown in the AlamarBlue assays. Interestingly, a direct cytotoxicity action of UA on amastigotes was also discarded in the TUNEL and TEM assays, indicating that the action of UA was executed by mechanisms other than apoptotic cell death. In contrast, UA was highly cytotoxic on the infective trypomastigote forms with an EC50 around half of the working concentration (10 μM). In agreement with these data, Uchiyama and colleagues showed a significant reduction in the parasitemia peak in *T. cruzi*-infected mice treated with UA (da Silva Ferreira et al., 2013). Like the amastigotes, epimastigotes, the extracellular replicative form of *T. cruzi*, displayed high resistance to the UA treatment with an IC50 that is ten times higher than that of the working concentration. These data showed the importance of testing compounds at the distinct biological forms of a pathogen given the possible different susceptibilities of these forms to the compound studied, as in the case of *T. cruzi.* Similar results have been observed in the treatment of *Leishmania donovani* with a semi-purified fraction of the wild mushroom *Grifola frondosa* (Sultana et al., 2018).

In our system, we demonstrated that UA induces autophagy in both macrophages and cardiac cells and that this response was functional due to the localization of acidic and hydrolytic markers on autophagosomes formed in the presence of the drug. We also observed that LC3 is recruited to amastigotes at the same time as LysoTracker staining, evidencing the connection of parasites with autolysosomes in cells treated with UA. Moreover, inhibition of autophagy with Spautin-1, the specific inhibitor of the PI3K of autophagy, interferes with the action of UA on amastigotes. In agreement with this, UA is less active to clear amastigotes in the autophagy-deficient macrophages obtained from Beclin-1 heterozygous knockout mice. Altogether, these data demonstrate that enhanced autophagy response occurred in the presence of UA, the most important mechanism in the elimination of *T. cruzi* amastigotes by the process of xenophagy. Other intracellular pathogens, such as *Mycobacterium tuberculosis* (Chai et al., 2019) and *Salmonella* (Ammanathan et al., 2019), were also eliminated by xenophagy. We conclude that UA could be a good candidate for the treatment of CD alone or in combination with the current therapies.

## Data Availability Statement

The original contributions presented in the study are included in the article/**Supplementary Material**. Further inquiries can be directed to the corresponding authors.

## Author Contributions

MV has contributed with the design, execution and analysis of experiments. She also contributed in the writing of the manuscript. SM, LM, BS, and JG collaborated in the design, performance and analysis of experiments. PR has contributed to the design of experiments and analysis of results. Also in the writing of the manuscript. All authors contributed to the article and approved the submitted version.

## Funding

This work has been partly supported by grants from Secretaría de Ciencia, Técnica y Posgrado (Sectyp, Universidad Nacional de Cuyo) and Agencia Nacional de Promoción Científica y Tecnológica (PICT# 2017 1456) to PR.

## Conflict of Interest

The authors declare that the research was conducted in the absence of any commercial or financial relationships that could be construed as a potential conflict of interest.

## Publisher’s Note

All claims expressed in this article are solely those of the authors and do not necessarily represent those of their affiliated organizations, or those of the publisher, the editors and the reviewers. Any product that may be evaluated in this article, or claim that may be made by its manufacturer, is not guaranteed or endorsed by the publisher.

## Supplementary Material

Supplementary Figure 1 Flow cytometry of Bone Marrow-derived Macrophages typing. **(A):** Population of macrophages obtained. **(B):** Doublet Elimination. **C** and **D:** Histograms show relative fluorescence intensity of CD11b-APC and F480 PERCP CY5 staining respectively.

Supplementary Figure 2 Alamar blue assay to assess UA toxicity in the cell models studied. RAW macrophages and H9C2 cells were treated for 24 h with 10 µm of UA and then cell viability was evaluated with Alamar blue reagent. Quantification of fluorescence intensity at 540 nm. Data represent the mean ± SEM of at least three independent experiments.

Supplementary Figure 3 Number of amastigotes in bone marrow macrophages of WT and Beclina KD mice. Bone marrow macrophages obtained from C57 wt and Beclin-1 KD mice (deficient in the Beclin-1 protein that participates in the autophagic pathway) were infected with trypomastigotes Y strain of *T. cruzi* (MOI=10) for 24 h and then the cells were washed and incubated in control medium alone or with 10 µM UA. The graph shows the quantification of the average number of amastigotes per cell ± SEM of four experiments.
