# Supplementary figures and images for: Induction of Autophagy by Ursolic Acid Promotes the Elimination of Trypanosoma cruzi Amastigotes From Macrophages and Cardiac Cells
Source: Front Cell Infect Microbiol. 2022 Jul 8;12:919096. doi: 10.3389/fcimb.2022.919096 (PMC9394444; doi:10.3389/fcimb.2022.919096)

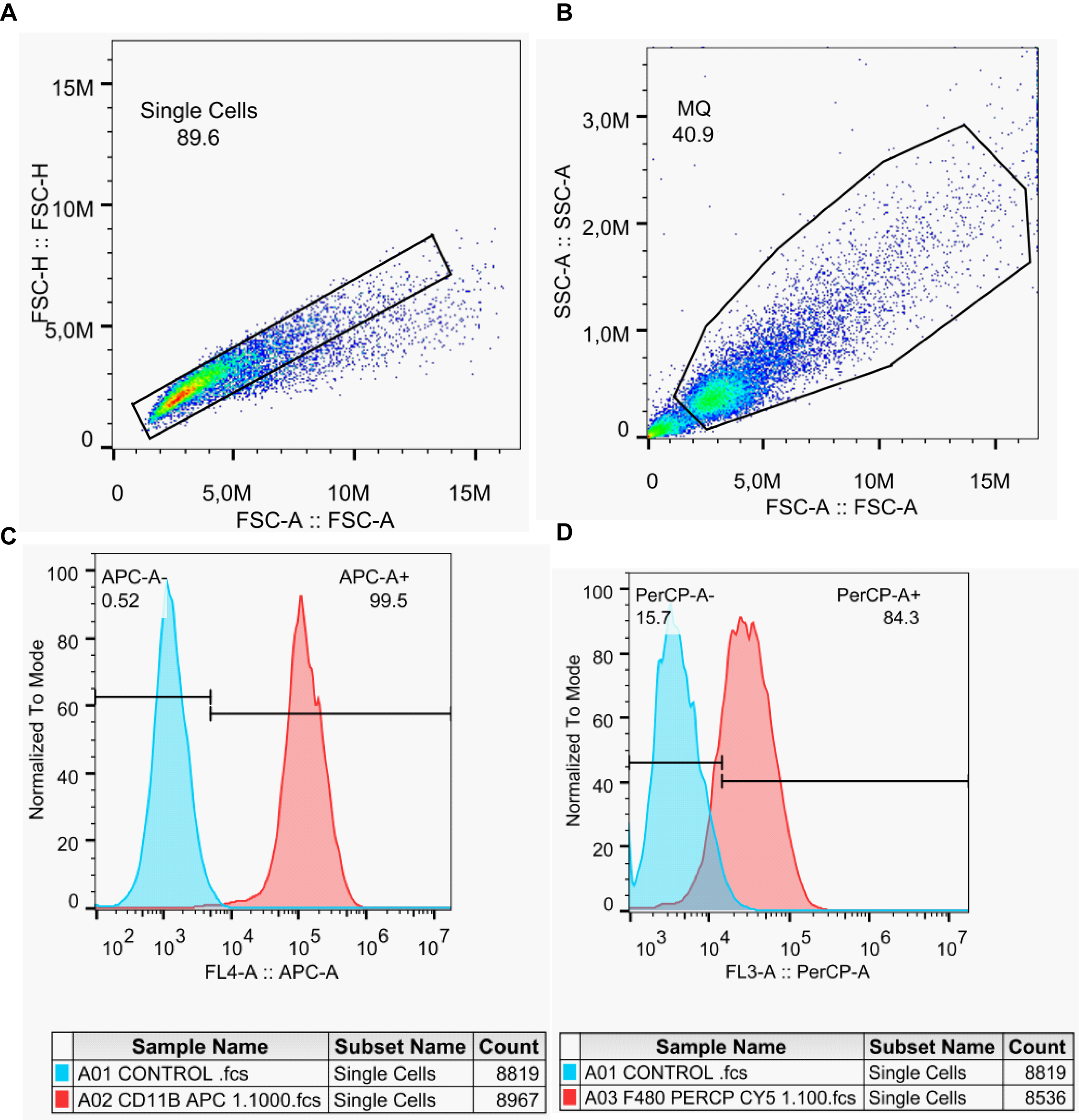

Supplement: Supplementary Figure 1 — Flow cytometry of Bone Marrow-derived Macrophages typing. (A): Population of macrophages obtained. (B): Doublet Elimination. C and D: Histograms show relative fluorescence intensity of CD11b-APC and F480 PERCP CY5 staining respectively. [file Image_1.tif]

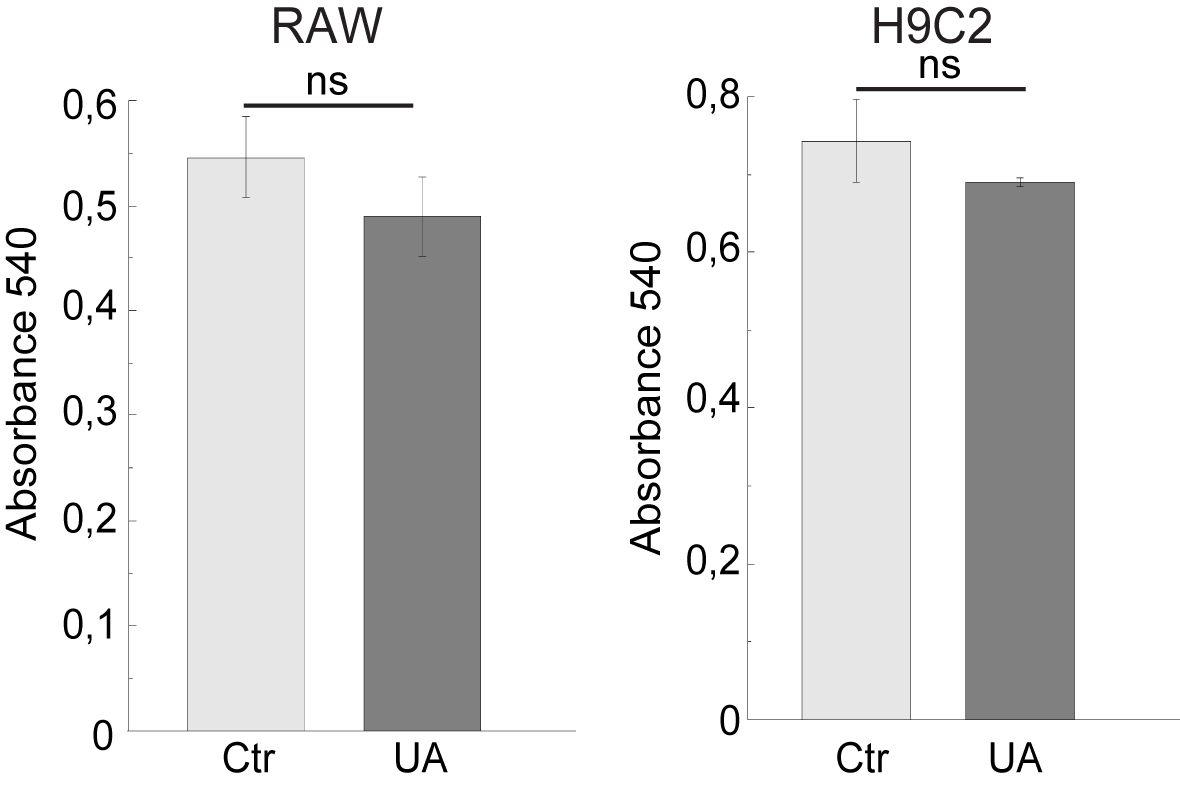

Supplement: Supplementary Figure 2 — Alamar blue assay to assess UA toxicity in the cell models studied. RAW macrophages and H9C2 cells were treated for 24 h with 10 µm of UA and then cell viability was evaluated with Alamar blue reagent. Quantification of fluorescence intensity at 540 nm. Data represent the mean ± SEM of at least three independent experiments. [file Image_2.tif]

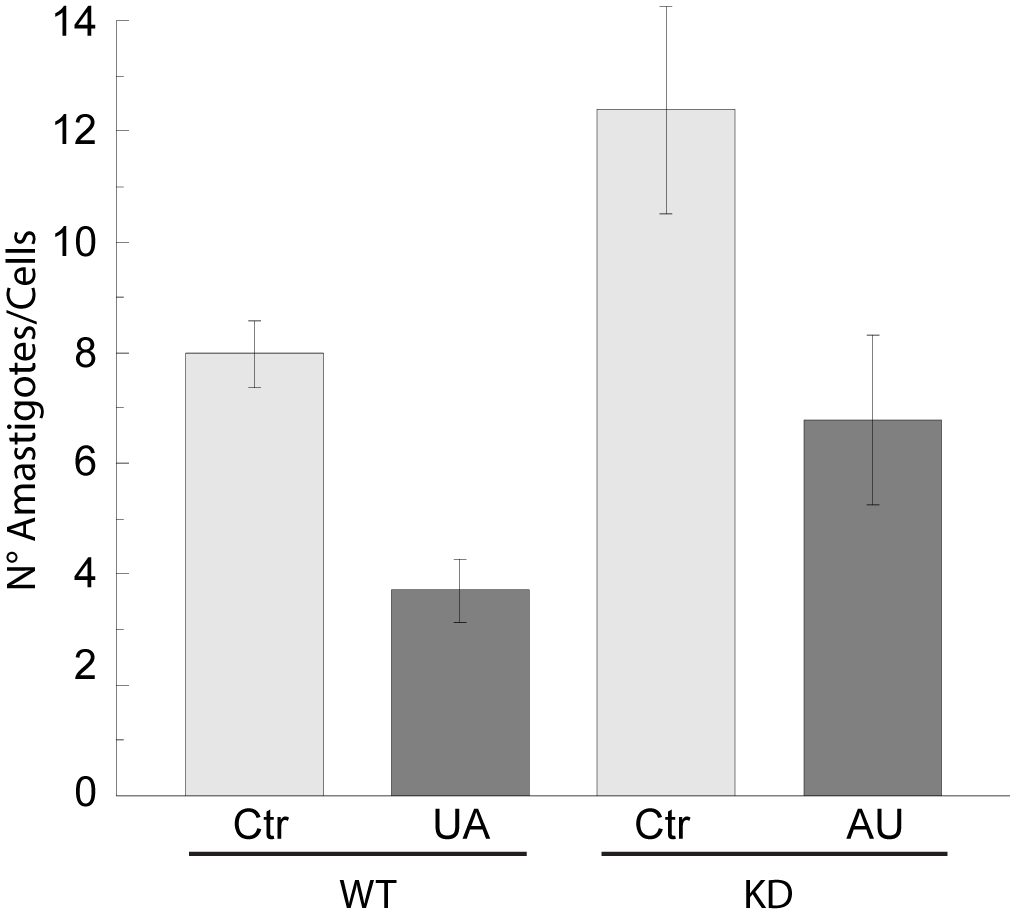

Supplement: Supplementary Figure 3 — Number of amastigotes in bone marrow macrophages of WT and Beclina KD mice. Bone marrow macrophages obtained from C57 wt and Beclin-1 KD mice (deficient in the Beclin-1 protein that participates in the autophagic pathway) were infected with trypomastigotes Y strain of T. cruzi (MOI=10) for 24 h and then the cells were washed and incubated in control medium alone or with 10 µM UA. The graph shows the quantification of the average number of amastigotes per cell ± SEM of four experiments. [file Image_3.tif]
